# Biomass-derived hard carbon host with added commercial silicon for high-capacity lithium-ion battery anodes

**DOI:** 10.1039/d5na01100k

**Published:** 2026-02-23

**Authors:** Alireza Fereydooni, Chenghao Yue, Puritut Nakhanivej, Maria Balart Murria, Mingrui Liu, Yuexi Zeng, Zhijie Wei, Qiuju Fu, Xuebo Zhao, Melanie J. Loveridge, Yimin Chao

**Affiliations:** a National Energy Key Laboratory for New Hydrogen-Ammonia Energy Technologies, Foshan Xianhu Laboratory Foshan 528200 P. R. China; b School of Chemistry, University of East Anglia Norwich UK Y.Chao@uea.ac.uk; c Tyndall Center for Climate Change Research, University of East Anglia Norwich UK; d Warwick Manufacturing Group (WMG), University of Warwick Coventry UK; e Shandong Provincial Key Laboratory of Chemistry Energy Storage and Novel Cell Technology, School of Materials Science and Engineering, Qilu University of Technology (Shandong Academy of Science) Jinan 250353 China

## Abstract

Silicon–carbon composites were prepared by introducing commercial silicon powder into a barley husk (BH)-derived SiO_2_/C hard-carbon host, producing Si–SiO_2_–C hybrid anodes with controlled Si loadings (20–50 wt%). Structural integration of Si within the porous BH matrix enabled mixed Li-storage behaviour, combining hard-carbon adsorption/pore filling with silicon alloying/dealloying. Increasing Si content raised reversible capacity but increased polarisation and accelerated capacity fade, indicating a trade-off between active Si utilisation and mechanical/electrochemical stability. At C/5 (defined relative to each anode's theoretical capacity), BH50–Si20, BH35–Si35 and BH20–Si50 delivered approximately ∼670, ∼880 and ∼1180 mAh g^−1^ after 50 cycles, respectively, compared with ∼380 mAh g^−1^ for BH and ∼350 mAh g^−1^ for graphite under the same protocol. Among the hybrids, BH35–Si35 provided the most balanced behaviour, combining high initial coulombic efficiency (∼87%) with stable voltage/d*Q*/d*V* signatures indicative of moderated silicon-driven degradation. A BH20–Si50//NMC622 full cell delivered 165 mAh g^−1^ (cathode basis) with 98.3% initial coulombic efficiency and retained 89% capacity after 100 cycles at C/5, demonstrating compatibility with a high-voltage layered cathode and practical energy-density potential.

## Abstract 2

Mechanical fusion embeds commercial Si (20–50 wt%) in barley-husk SiO_2_/C hard carbon, boosting capacity while limiting resistance growth. BH20–Si50//NMC622: 165 mAh g^−1^, 98.3% ICE, and 89% retention (100 cycles).

## Graphical abstract

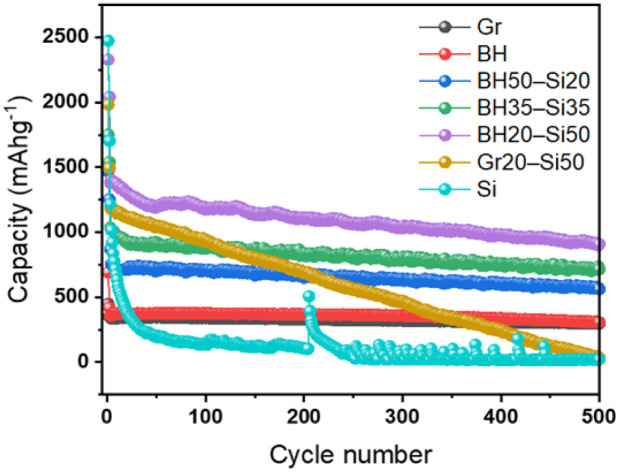

## 1. Introduction

The growing demand for lithium-ion batteries (LIBs) in electric vehicles, portable electronics, and grid-scale storage systems continues to drive the search for anode materials that combine high energy density with long-term cycling stability.^1,2^ Currently, graphite is the most widely used commercial anode due to its excellent structural reversibility and moderate lithiation potential.^3,4^ However, its relatively low theoretical capacity of 372 mAh g^−1^ imposes a significant limitation on the overall energy density of next-generation LIBs.^5^ Silicon, in contrast, offers a much higher theoretical capacity of ∼4,200 mAh g^−1^ based on the formation of Li_22_Si_5_, making it an attractive alternative for high-capacity anode design.^6,7^

Due to its remarkable theoretical capacity, silicon is widely considered a leading candidate for next-generation anodes. However, practical deployment remains restricted by severe volume changes during alloying–dealloying, which promote particle fracture, electrical disconnection, and repeated solid–electrolyte interphase (SEI) reformation.^8,9^ Biomass-derived carbon–silicon composites have therefore gained attention because carbon frameworks can provide both electronic percolation and mechanical buffering, improving cycling stability when silicon is appropriately integrated within a compliant carbon matrix.^10–12^ Recent literature summarises a broad range of biomass precursors and compositing routes, while emphasising that long-term stability is governed by the balance between silicon loading, microstructural accommodation space, and interfacial robustness.^13^

In response, biomass-derived carbon–silicon composites have therefore gained attention because carbon frameworks can provide both electronic percolation and mechanical buffering, improving cycling stability when silicon is appropriately integrated within a compliant carbon matrix.^14^ Recent literature summarises a broad range of biomass precursors and compositing routes, while emphasising that long-term stability is governed by the balance between silicon loading, microstructural accommodation space, and interfacial robustness.^15,16^

Beyond electrochemical performance, biomass-derived electrodes have attracted increasing interest as part of a wider move towards greener battery components and improved circularity of energy-storage materials.^17^ Critical assessments of sustainable battery manufacturing highlight biomass-derived anodes as a particularly promising option, provided that scalable processing routes are adopted and performance is validated under realistic cycling conditions.

Several reports have demonstrated that integrating silicon with porous carbon frameworks derived from sources such as rice husks,^18^ corn stalks,^19^ or coconut shells^20^ can enhance cycle stability by retaining structural integrity and promoting stable SEI formation. However, the diversity of biomass feedstocks and the variability in their carbon–silicon interaction mechanisms highlight the need for systematic investigations into optimized hybrid architectures.

In our previous work, we introduced barley husks as a viable biomass precursor for producing hard carbon anodes in LIBs.^21^ That study focused on understanding the structural, compositional, and electrochemical properties of barley husk-derived carbon relative to commercial graphite. Comprehensive material characterization revealed a predominantly amorphous carbon matrix with embedded silica phases and a moderate specific surface area. These features contributed to enhanced Li storage sites and favourable SEI formation. Electrochemical tests confirmed that barley husk-based anodes could deliver higher initial capacity and improved rate capability compared to graphite, owing to their disordered structure and hierarchical porosity.^21^ While that work established barley husks as a sustainable and structurally versatile carbon source, the material was not optimized for high-capacity applications, nor was its potential as a mechanical buffer for silicon explored.

Despite the growing interest in silicon–carbon composites, most prior studies have focused on synthetic carbons or commercial graphite as the host matrix, with limited emphasis on integrating high-silicon content into biomass-derived systems. While some biomass carbons have been explored in hybrid configurations, their role has largely been restricted to passive conductivity enhancement or morphological templating, without systematic investigation into their structural compatibility with silicon.^22–24^ In particular, the capacity of natural biomass carbons to mitigate the severe mechanical stress and interfacial instability associated with silicon lithiation remains underexplored. Furthermore, few reports address the design of carbon–silicon hybrids where the biomass matrix inherently contains inorganic constituents—such as silica—that could contribute to mechanical buffering or improved adhesion between components.^25–27^ In our earlier study,^21^ the silica-rich nature of barley husk-derived carbon was identified but not fully leveraged. This raises an important question: can the structural and chemical characteristics of barley husks be exploited not only as a carbon source but also as a stabilizing framework for high-loading silicon anodes? To date, no systematic effort has been made to evaluate the electrochemical performance of barley husks and silicon hybrid anodes across varying compositions, or to assess their full-cell integration potential against industry-relevant cathodes.

In this study, rather than pursuing complex chemical bonding strategies, we explore a straightforward engineering route to use barley husk-derived hard carbon as a functional host for silicon in high-performance LIB anodes. Building on the structural insights established in our previous work, we design a series of hybrid anodes *via* a cost-effective ball-milling approach, precisely optimising the mass ratios to leverage the natural silica-rich framework of barley husks. The goal is to determine whether the intrinsic features of barley husks—such as their disordered carbon structure, hierarchical porosity, and embedded silica—can contribute to mitigating silicon's volumetric expansion effects on the anode, thereby enhancing cycling stability and rate performance. We conduct comprehensive electrochemical testing, including half-cell and full-cell evaluations against NMC622 cathodes, to assess capacity retention, coulombic efficiency, and rate capability across different hybrid compositions. The fabrication method involves only carbonization and mechanical mixing, emphasizing simplicity and scalability. Our findings demonstrate that barley husk-based hybrid anodes not only outperform graphite and pure silicon anodes but also offer a practical route toward sustainable, high-capacity anode design for next-generation LIBs.

## 2. Results and discussion

The low-magnification SEM image of Si powder in Fig. S2 shows agglomerated, irregularly shaped particles forming a loosely packed network. Higher-magnification images combined with EDS elemental maps in Fig. S2b–f reveal that these agglomerates consist of densely packed Si domains coated by a thin conductive Au layer (used for imaging), with only a weak, diffuse O signal. The absence of pronounced compositional inhomogeneity in the Si maps indicates that the powder is chemically uniform at the micrometre scale.

Dynamic light scattering (DLS) measurements reported in Fig. S3a indicate a broad particle-size distribution concentrated between approximately 0.3 and 2 µm, with a tail extending to around 5 to 6 µm. These values are larger than the nominal primary particle size specified by the supplier, reflecting the presence of weakly bound agglomerates in the suspension. XRD analysis of the commercial Si powder (Fig. S3b) displays a series of sharp diffraction peaks at 2*θ* ≈ 28°, 47°, 56°, 69° and 76°, consistent with crystalline diamond-cubic Si, according to Joint Committee Diffraction Standard (JCPDS): 96-901-3104.^28^ No additional crystalline reflections attributable to SiO_2_ are observed, indicating that any surface oxide is either amorphous or below the detection limit. This assignment is reinforced by the combined XRD comparison in Fig. S4, where the same Si reflections are clearly retained in the BH–Si and Gr–Si composite electrodes, while the BH-derived host exhibits a broad amorphous carbon contribution rather than sharp graphitic staging peaks. The combined XRD comparison confirms that these crystalline Si reflections are retained in the BH–Si and graphite–Si composite electrodes, consistent with successful incorporation of the Si phase into the hybrid coatings.

**Fig. 1:**
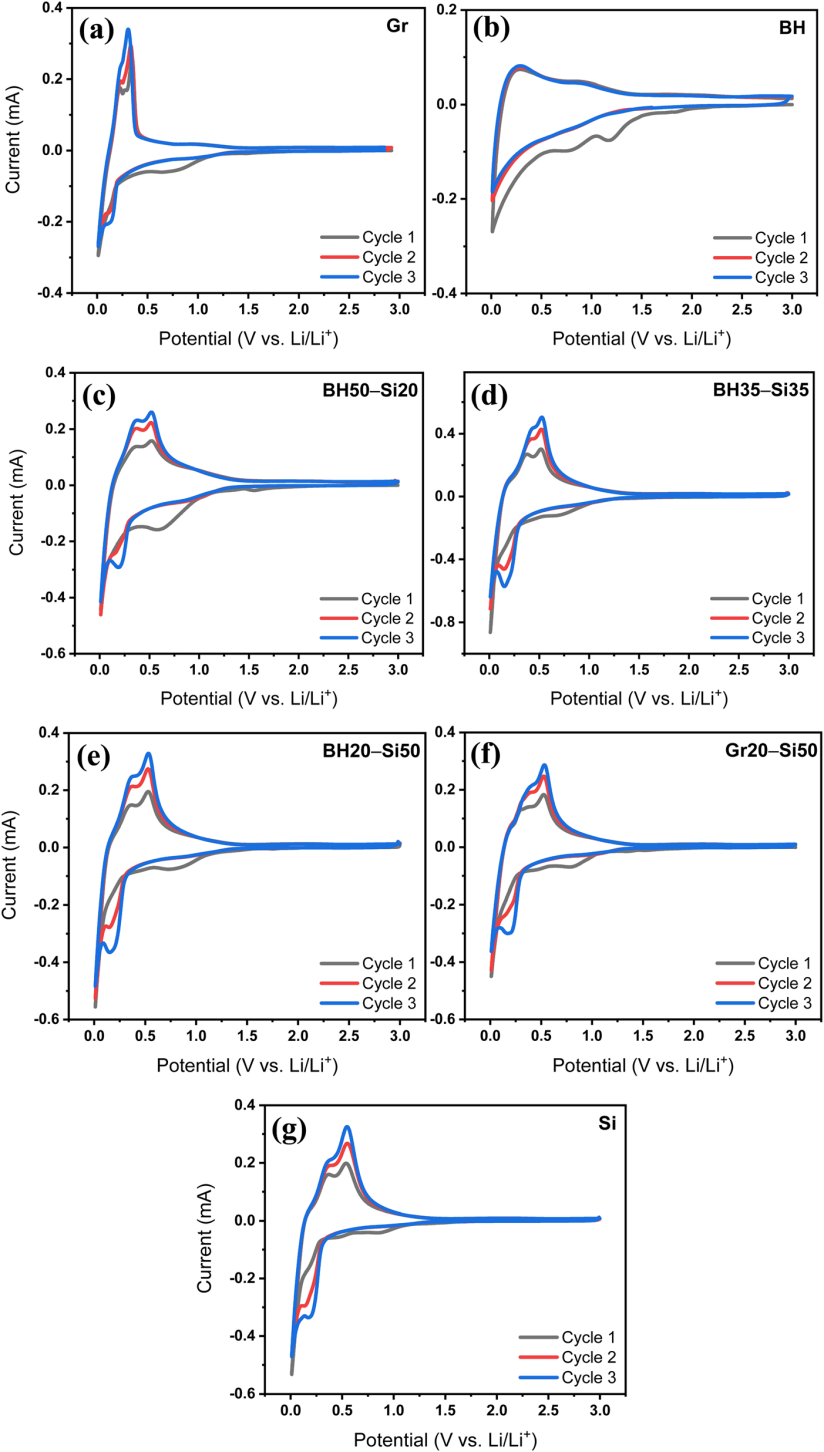
CV curves of (a) graphite (Gr), (b) pure barley husk (BH), (c) BH50–Si20, (d) BH35–Si35, (e) BH20–Si50, (f) Gr20–Si50, and (g) pure silicon (Si) anodes recorded at a scan rate of 0.2 mV s^−1^ in the voltage range of 3.0–0.01 V *vs.* Li/Li^+^ during the first three cycles with identical mass loading across the electrodes.

The electrochemical behaviour of all anodes was initially assessed using CV analysis, as shown in Fig. 1. Each anode exhibits distinct lithiation/delithiation features depending on its composition, offering insight into the underlying charge storage mechanisms and reaction reversibility. The CV profile of the Gr anode in Fig. 1a exhibits electrochemical features typical of Li^+^ intercalation into graphitic carbon. During the first cathodic sweep, a broad reduction peak centred at around 0.6–0.8 V *vs.* Li/Li^+^ is observed, which is absent in subsequent cycles. This peak corresponds to the formation of a solid electrolyte interphase (SEI) resulting from the reductive decomposition of the electrolyte components on the graphite surface. The disappearance of this peak in the following cycles indicates the development of a passivating SEI layer that stabilizes the electrode–electrolyte interface, a phenomenon known to contribute to the initial irreversible capacity loss in graphite anodes.^29^ As the potential further decreases, a sharp cathodic peak below 0.25 V *vs.* Li/Li^+^ appears, corresponding to stage-wise Li^+^ intercalation into graphite layers, culminating in the formation of the LiC_6_ phase. This is followed by a narrow anodic peak at ∼0.2–0.25 V *vs.* Li/Li^+^ in the reverse sweep, associated with the delithiation of LiC_6_ back to graphite.^29,30^ The symmetry and consistency of these intercalation/deintercalation peaks across the second and third cycles confirm the high reversibility and stable kinetics of Li storage in graphite. This behaviour is characteristic of well-ordered graphitic structures and reflects minimal polarization and effective SEI passivation.

The BH anode (Fig. 1b) exhibits a distinctly different electrochemical profile compared to graphite, reflecting the behaviour of disordered hard carbon. The first cathodic scan shows a broad hump between ∼1.0 and 0.2 V *vs.* Li/Li^+^, which is associated with surface-controlled Li storage, including SEI formation, Li^+^ adsorption at defect sites, and interactions with surface functionalities and residual silica phases.^31^ A subtle shoulder near 0.4–0.6 V *vs.* Li/Li^+^ corresponds to partial reduction of surface oxygen groups or interactions with the embedded SiO_2_ content. Below ∼0.2 V *vs.* Li/Li^+^, the current gradually increases in a sloping fashion, indicating Li^+^ insertion into pores and disordered carbon domains, rather than graphitic staging transitions. On the anodic sweep, a broad delithiation feature appears at around 0.2–0.5 V *vs.* Li/Li^+^, stabilizing after the first cycle, which confirms improved interfacial kinetics and SEI maturation from cycle 2 onward.^21,32^

Upon introducing silicon into the BH matrix, a progressive evolution in the CV features is observed (Fig. 1c–e). The BH50–Si20 hybrid anode (Fig. 1c), with a lower Si content, retains much of the broad BH-like lithiation profile while beginning to exhibit a distinct cathodic peak below 0.1 V *vs.* Li/Li^+^, characteristic of Si alloying with Li (Li_*x*_Si formation).^33^ This new peak confirms the electrochemical activity of the silicon component, while the overall smoother profile and broader anodic feature suggest that the BH matrix continues to buffer and regulate the reaction environment.

As the Si content increases to 35 wt% and 50 wt% in BH35–Si35 and BH20–Si50 anodes (Fig. 1d and e), respectively, the intensity of the sharp lithiation peak below 0.1 V *vs.* Li/Li^+^ significantly increases and the anodic scan develops more pronounced features at around 0.35–0.55 V *vs.* Li/Li^+^, corresponding to the stepwise dealloying of Li_*x*_Si species. Contributions from the BH matrix remain evident as broad current responses between 0.2 and 0.6 V *vs.* Li/Li^+^. This overlapping behaviour suggests that both components remain electrochemically active and that the BH matrix continues to modulate the volume change and charge distribution during cycling. Notably, the cathodic and anodic peaks in BH35–Si35 anode are sharper and better defined than those in the BH50–Si20 anode, indicating enhanced Si utilization, while still retaining cycling stability—likely due to the optimal balance between mechanical buffering and active capacity. In contrast, the Gr20–Si50 anode (Fig. 1f), which contains the same Si content as the BH20–Si50 anode, shows more polarized and asymmetric peaks, particularly in the first cycle. Sharp lithiation and delithiation features below 0.1 V *vs.* Li/Li^+^ and above 0.4 V *vs.* Li/Li^+^ are present, but the broader separation between anodic and cathodic peaks suggests higher overpotential and lower reaction reversibility. This behaviour implies that graphite is less effective than BH in stabilizing the Si network, likely due to its less porous (graphite: 69.254 Å ≈ 6.93 nm; BH: 51.844 Å ≈ 5.18 nm), less compliant structure, which is prone to interfacial instability and SEI breakdown.

Finally, the pure Si anode (Fig. 1g) shows the most pronounced and narrow lithiation peak below 0.1 V *vs.* Li/Li^+^, along with steep delithiation peaks between 0.35 and 0.55 V *vs.* Li/Li^+^. However, the rapid drop in current intensity and the peak shift across the cycles indicate significant SEI formation, electrode degradation, and Li trapping, all of which are well-documented limitations of Si anodes.^34^ Compared to this baseline, the BH–Si hybrid anodes clearly demonstrate a more gradual, stabilized electrochemical response, particularly in the BH35–Si35 anode, which balances capacity, reversibility, and kinetic accessibility.

**Fig. 2:**
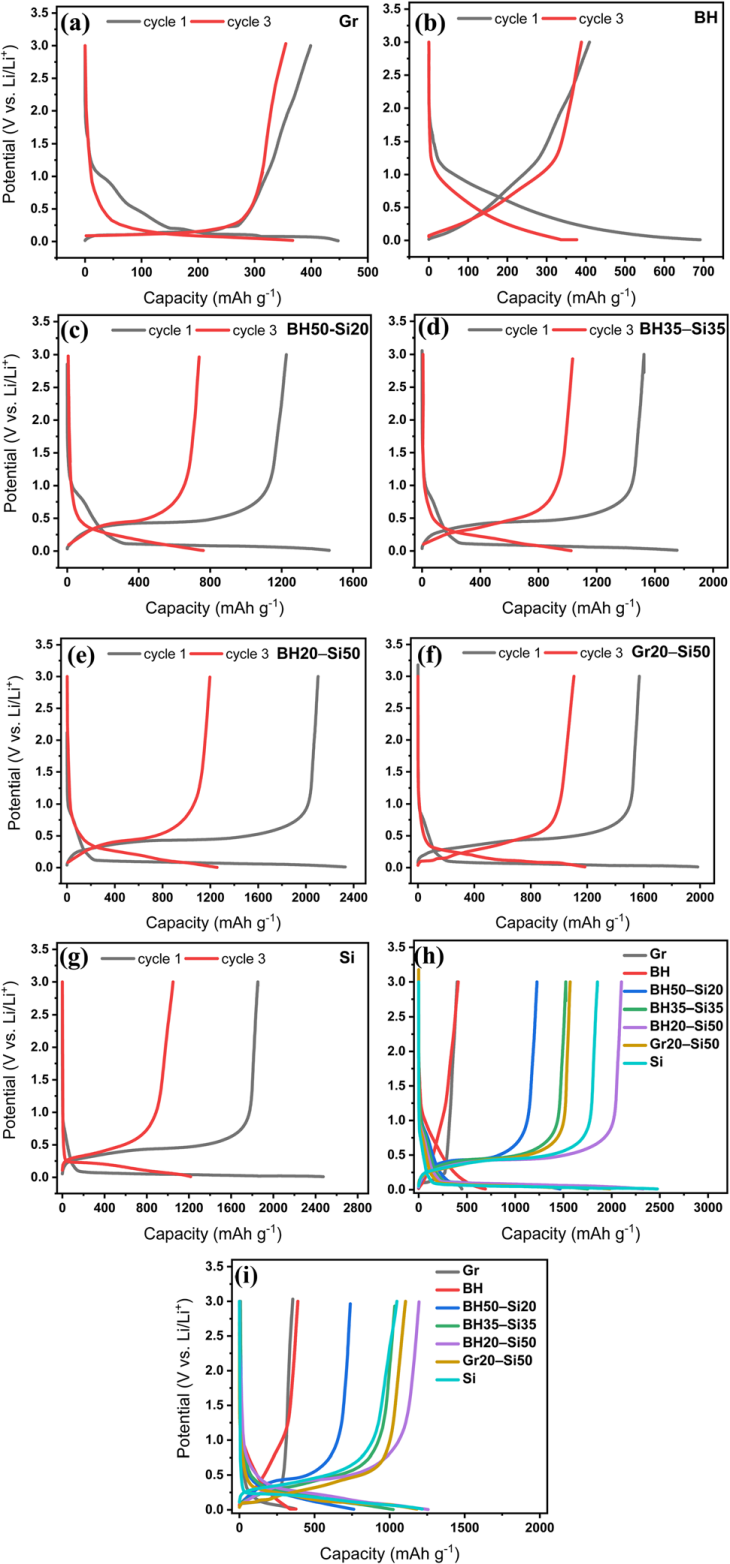
Galvanostatic charge–discharge voltage profiles of (a) graphite (Gr), (b) pure barley husk (BH), (c) BH50–Si20, (d) BH35–Si35, (e) BH20–Si50, (f) Gr20–Si50, and (g) pure silicon (Si) anodes, showing the 1st and 3rd cycles. (h and i) Comparative overlays of all anode types in the 1st and 3rd cycles, respectively. All profiles are recorded within the voltage range of 0.01–3.0 V *vs.* Li/Li^+^ and at a current rate of C/5 (74–360 mA g^−1^ depending on anode formulation; see Fig. S1 for exact values).

Galvanostatic charge–discharge profiles at C/5 are reported for the first and third cycles, as shown in Fig. 2. The Gr anode (Fig. 2a) displays a well-defined discharge plateau below 0.2 V *vs.* Li/Li^+^ and a sharp charge plateau at ∼0.25 V *vs.* Li/Li^+^, corresponding to Li^+^ intercalation and deintercalation into graphite layers. The narrow voltage hysteresis (∼0.07 V) and stable profile across cycles reflect efficient charge transfer and high reversibility, consistent with its low irreversible capacity loss (∼11%) and initial coulombic efficiency (ICE) of ∼89%. The BH anode (Fig. 2b) however, exhibits a sloped voltage profile with no distinct plateaus, spanning the entire 0.01–1.0 V *vs.* Li/Li^+^ range. This behaviour aligns with its broad CV response and reflects Li storage *via* surface interaction, defect trapping, and pore filling. The lower ICE (∼60%) and absence of sharp features point to initial SEI formation and capacitive behaviour, typical of hard carbon.

Upon Si addition, the hybrids show evolving voltage characteristics. The BH50–Si20 anode (Fig. 2c) retains BH's sloping profile but begins to exhibit a weak lithiation plateau near 0.1 V *vs.* Li/Li^+^, confirming partial Si activation. BH35–Si35 and BH20–Si50 anodes (Fig. 2d and e, respectively) develop increasingly pronounced plateaus below 0.1 V *vs.* Li/Li^+^ and broad delithiation features at 0.35–0.55 V *vs.* Li/Li^+^, consistent with Li_*x*_Si alloying/dealloying. The BH35–Si35 anode achieves a balanced behaviour, with an ICE of ∼87% and moderate hysteresis (∼0.12 V *vs.* Li/Li^+^), suggesting improved Si utilization while preserving BH's buffering role. In the BH20–Si50 anode, the increased Si content enhances capacity but also introduces higher polarization and a slight drop in CE (∼80%). The Gr20–Si50 anode (Fig. 2f) shows sharper plateaus but greater hysteresis (∼0.18 V *vs.* Li/Li^+^) than the BH20–Si50 anode, reflecting sluggish kinetics and interfacial stress. This matches the CV observation of broader peak separation and underscores graphite's limited capacity to buffer Si expansion.

Pure Si (Fig. 2g) shows the steepest and most defined lithiation/delithiation plateaus but suffers from severe capacity loss by the third cycle and the lowest ICE (∼72%), indicative of unstable SEI growth and Li trapping. Overlaid profiles (Fig. 2h and i) summarize the performance spectrum: Gr and BH anodes at either end with stable but modest profiles, Si with high but fading capacity, and BH–Si hybrid anodes, especially BH35–Si35, offering a favourable compromise between capacity, reversibility, and structural stability.

**Fig. 3:**
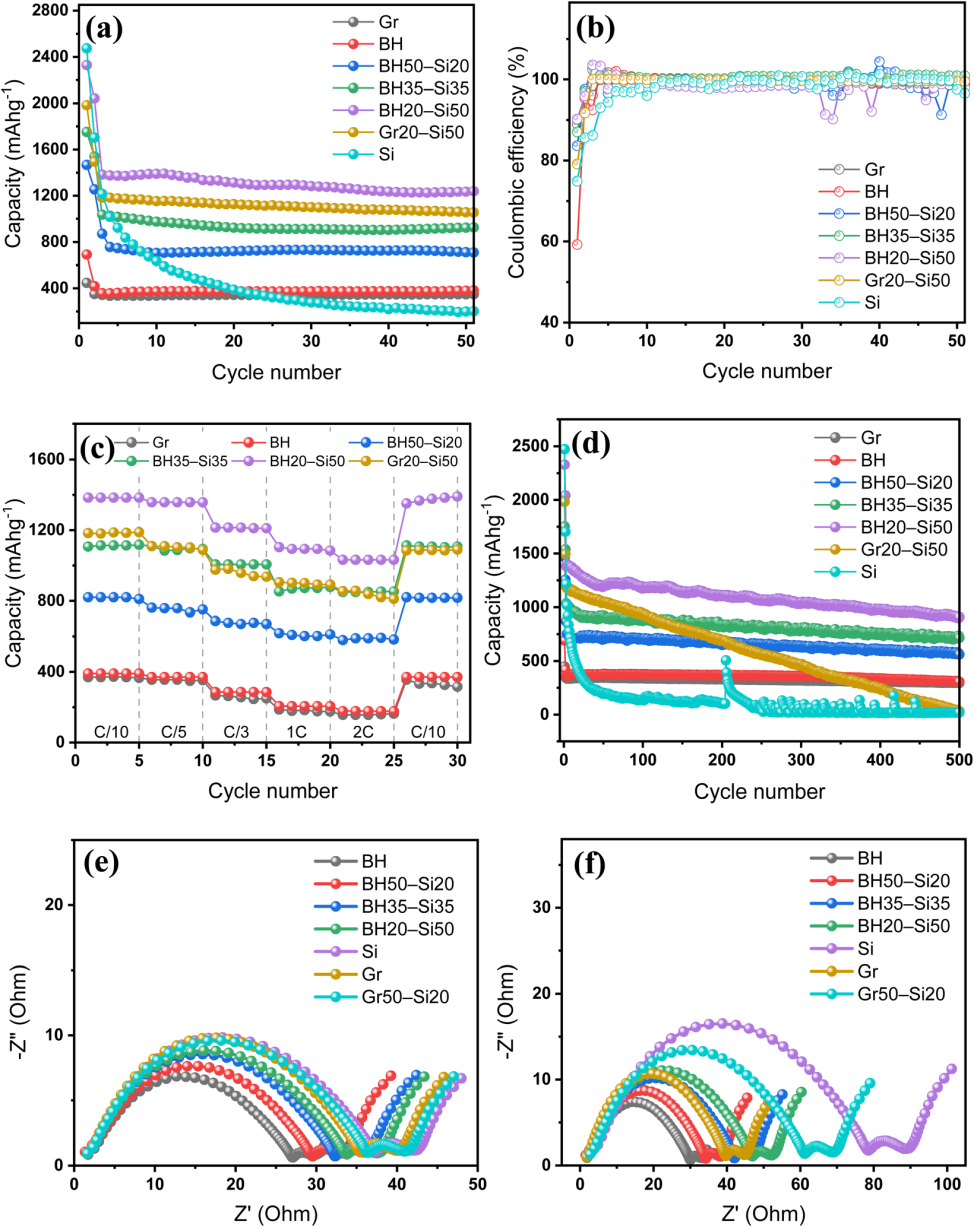
(a) Cycling performance of all anodes at a current rate of C/5 over 50 cycles. (b) Corresponding coulombic efficiency (CE) profiles during cycling. (c) Rate capability of selected anodes evaluated across multiple current densities (C/10 to 2C) and returned to C/10. (d) Long-term cycling performance of all anodes over 500 charge–discharge cycles at a current rate of C/5. (e) Nyquist plots measured after the 1st cycle and (f) after the 50th cycle, comparing interfacial resistance evolution across all anode formulations.

The cycling stability and rate capability results shown in Fig. 3 further validate the electrochemical behaviour inferred from CV (Fig. 1) and voltage profiles (Fig. 2). At a constant current rate of C/5, the cycling performance (Fig. 3a) highlights substantial differences in both capacity and stability across the electrode formulations. Graphite and BH deliver stable but moderate capacities of ∼350 mAh g^−1^ and ∼380 mAh g^−1^, respectively. For graphite, the reversible capacity is fundamentally limited by the LiC_6_ stoichiometry (372 mAh g^−1^) associated with Li^+^ intercalation into graphitic layers, which also underpins its stable cycling under modest volume change. For BH-derived hard carbon, the sloping lithiation behaviour and slightly higher capacity are consistent with additional Li storage *via* adsorption at defects/heteroatom sites and pore filling within disordered carbon domains, while the absence of large alloying-type strain supports good capacity retention.^35,36^ Both anodes retain >95% of their capacity over 50 cycles, indicating good structural and interfacial stability under the applied conditions. This interpretation is supported by the rapid stabilisation of coulombic efficiency (Fig. 3b) and the limited impedance growth between the 1st and 50th cycles (Fig. 3e and f), consistent with a largely stabilised electrode–electrolyte interface.

Upon Si incorporation, the BH–Si hybrid anodes demonstrate enhanced capacities. The BH50–Si20 anode achieves ∼670 mAh g^−1^ after 50 cycles, while BH35–Si35 and BH20–Si50 anodes reach ∼880 mAh g^−1^ and ∼1180 mAh g^−1^, respectively. Despite increasing Si content, all BH–Si hybrid anodes retain good stability with gradual capacity fade and no sudden drops, showing that the BH matrix mitigates Si-induced volume changes and helps preserve electrode integrity during cycling. For instance, the capacity retention observed here is superior to that of several recently reported biomass-derived Si/C anodes. As frequently observed in recent studies,^37,38^ simple mechanical blends of silicon and biomass carbon typically suffer from rapid capacity decay due to the lack of effective buffering against particle pulverization. In contrast, Gr20–Si50, which has the same Si loading as BH20–Si50, shows faster capacity decay (∼960 to ∼880 mAh g^−1^), suggesting that graphite is less effective as a mechanical buffer. The pure Si anode initially achieves >2400 mAh g^−1^, which rapidly declines to <200 mAh g^−1^ by cycle 50—confirming its poor structural resilience.

Coulombic efficiency trends (Fig. 3b) provide additional insight. In the present study, all anodes stabilize above 98% within 10 cycles, indicating effective SEI formation. BH–Si hybrid anodes exhibit slightly delayed CE stabilization compared to graphite and BH, due to increased interfacial activity from Si. Among them, the BH35–Si35 anode reaches >99% by cycle 15 and retains consistent CE thereafter, highlighting its balance between capacity and interfacial stability.

Rate performance (Fig. 3c) further differentiates the anodes under dynamic operating conditions. The BH20–Si50 anode consistently delivers the highest capacity across all current rates, retaining ∼950 mAh g^−1^ even at 2C and recovering fully upon return to C/10. BH35–Si35 and BH50–Si20 anodes follow with intermediate capacities (∼820 mAh g^−1^ and ∼650 mAh g^−1^ at 1C, respectively), but all BH–Si hybrid anodes show excellent rate response and capacity recovery, confirming robust structural integrity and fast ion transport. In contrast, Gr20–Si50 shows diminished rate tolerance, while performing poorly at higher C-rates due to its diffusion-limited intercalation mechanism.

The long-term cycling stability of the anodes is shown in Fig. 3d. The pure Si anode rapidly loses capacity, dropping to negligible levels within the initial few cycles due to severe structural failure from extensive volume changes. In contrast, the Gr anode demonstrates excellent stability, retaining 96.6%, 94.4%, 90.9%, 87.7%, and 84.6% of its initial capacity after 100, 200, 300, 400, and 500 cycles, respectively. Similarly, the pure BH anode shows stable but moderate performance, with retention values of 97.9%, 96.4%, 93.9%, 88.0%, and 80.8% at these intervals, confirming its robust structural integrity.

For Si-containing BH composites, increasing Si content results in higher initial capacity but gradually decreases cycling stability. The BH50–Si20 anode shows notably stable cycling performance, retaining 97.6%, 90.3%, 87.2%, 82.7%, and 78.1% after each interval. The BH35–Si35 anode retains slightly lower values at 89.0%, 84.1%, 79.9%, 77.5%, and 72.9%, which remain commendable given the higher Si fraction. The BH20–Si50 anode, despite the highest Si content, retains reasonable stability with retention values of 86.3%, 80.5%, 75.6%, 71.3%, and 65.8%, highlighting the effective buffering capability of BH at substantial Si loadings. In sharp contrast, the Gr20–Si50 composite suffers from rapid capacity fading, with significant deterioration observed after each interval (67.6%, 49.8%, 33.2%, 17.2%, and ultimately just 2.4% after 500 cycles). This rapid decline underscores graphite's inadequacy in accommodating Si-induced volume expansion compared to BH-based matrices.

Electrochemical impedance spectra provide complementary evidence for this interfacial stability. After the 1st cycle (Fig. 3e), all electrodes show a depressed semicircle in the high-to-mid frequency region followed by a low-frequency diffusion tail, consistent with interfacial (SEI/charge-transfer) processes coupled to Li^+^ transport. By the 50th cycle (Fig. 3f), graphite and BH retain comparatively compact semicircles with only modest growth in impedance, indicating limited resistance build-up during cycling. In contrast, the pure Si electrode exhibits a pronounced enlargement of the semicircle and a substantial rightward shift of the spectrum, consistent with marked interfacial resistance growth associated with an unstable SEI and progressive loss of electrical contact during repeated alloying.

The comparatively restrained impedance growth for BH–Si hybrids suggests that the BH matrix retains interfacial contact and suppresses progressive electrical isolation of Si domains during cycling, consistent with more effective dispersion and buffering relative to graphite–Si and Si-only electrodes. Together, these results confirm that BH–Si hybrid anodes offer a compelling combination of high capacity, rate capability, and stability. In particular, BH35–Si35 emerges as the optimal formulation, balancing structural buffering, electrochemical reversibility, and performance retention under practical cycling conditions.

**Fig. 4:**
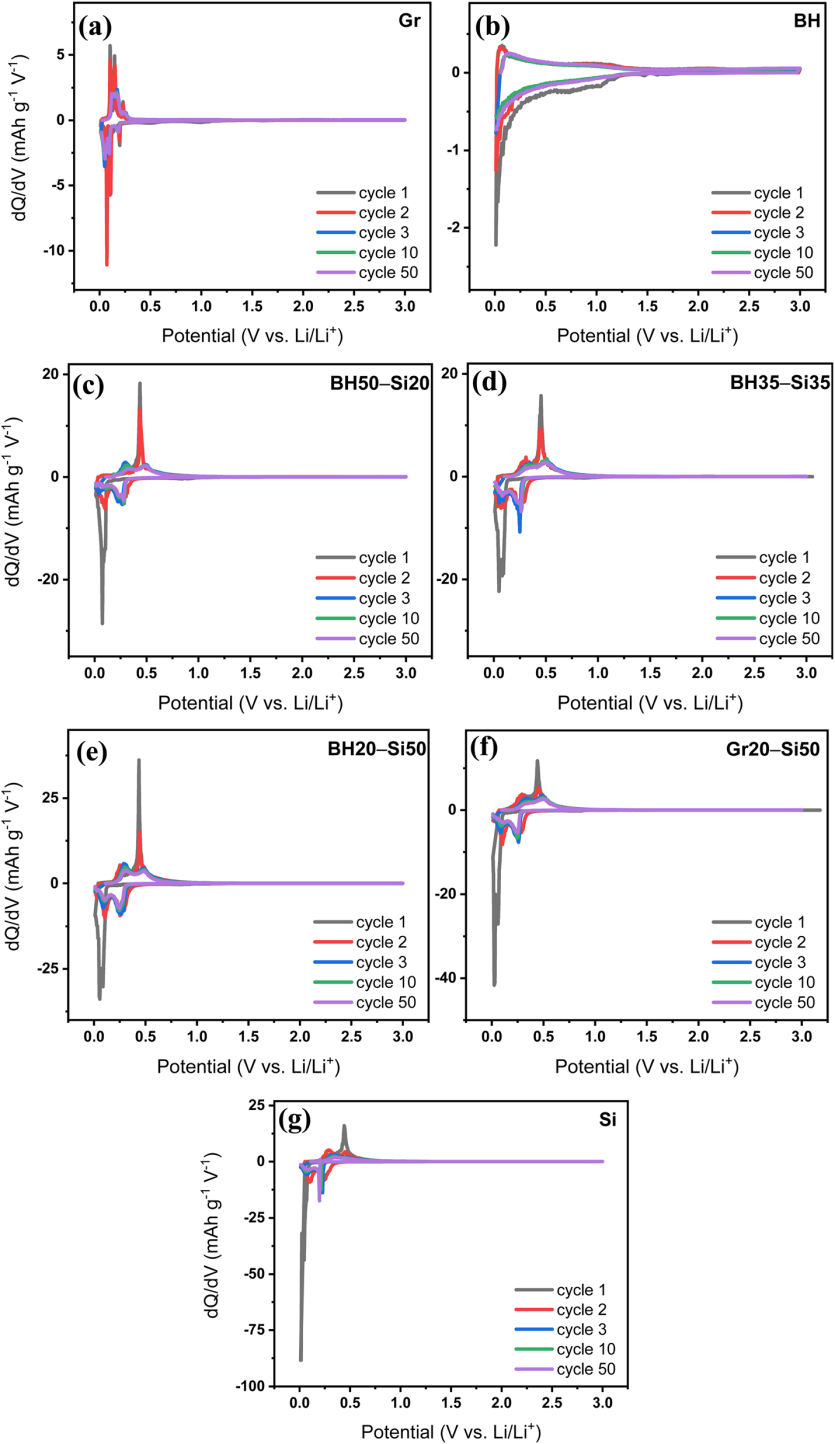
Differential capacity (d*Q*/d*V*) plots of (a) graphite (Gr), (b) barley husk (BH), (c) BH50–Si20, (d) BH35–Si35, (e) BH20–Si50, (f) Gr20–Si50, and (g) silicon (Si) anodes recorded at a current rate of C/5 over selected cycles (1st, 2nd, 3rd, 10th, and 50th cycles).

The differential capacity (d*Q*/d*V*) profiles in Fig. 4 provide further insight into the redox mechanisms and cycling stability of the anodes by tracking the evolution of lithiation/delithiation processes over time. For the Gr anode (Fig. 4a), sharp and well-defined peaks appear at ∼0.1 V and ∼0.25 V *vs.* Li/Li^+^ in the cathodic and anodic directions, respectively, corresponding to the reversible staging transitions between graphite and LiC_6_. These features remain highly stable across 50 cycles, confirming excellent reversibility and minimal degradation, in agreement with the consistent CV and voltage profiles observed earlier. In contrast, the BH anode (Fig. 4b) displays broad and diffuse d*Q*/d*V* features, with cathodic activity spread from ∼1.0 to 0.1 V *vs.* Li/Li^+^ and a similarly wide anodic region from ∼0.1 to 0.6 V *vs.* Li/Li^+^. These broad signals are indicative of pseudocapacitive and diffusion-limited Li^+^ storage within disordered carbon domains and pores. Importantly, the curves remain relatively stable from cycle 3 to 50, confirming the structural robustness and interfacial stability of the BH matrix.

For the BH–Si hybrid anodes, the d*Q*/d*V* profiles reveal the progressive influence of Si on the redox behaviour. The BH50–Si20 anode (Fig. 4c) shows both the broad BH-related features and emerging sharp peaks near ∼0.05 V (lithiation) and ∼0.4–0.5 V *vs.* Li/Li^+^ (delithiation), characteristic of Li_*x*_Si alloying/dealloying. These peaks intensify with increasing Si content in BH35–Si35 and BH20–Si50 anodes (Fig. 4d and e, respectively), where they become more pronounced and better defined. These hybrid systems retain reasonably stable peak positions through cycle 50, especially in the BH35–Si35 anode, which displays minimal peak broadening or shift—highlighting its balanced kinetics and structural integrity. In contrast, the BH20–Si50 anode begins to show slight peak smearing by cycle 50, suggesting moderate polarization growth likely due to higher silicon content and increasing mechanical stress.

Gr20–Si50 (Fig. 4f), which contains the same Si content as the BH20–Si50 anode but uses graphite as the host, shows more significant peak distortion and broadening over time, particularly in the anodic region. This degradation in signal definition implies less effective structural accommodation of silicon and greater instability at the graphite–Si interface. This correlates with the faster capacity fading and greater voltage hysteresis seen earlier. The pure Si anode (Fig. 4g) exhibits the highest peak intensity initially, with steep lithiation and delithiation peaks corresponding to multi-step Li_*x*_Si alloying transitions. However, these peaks quickly decay and broaden with cycling, accompanied by a noticeable drop in peak height by cycle 50. This confirms substantial SEI reformation, Li trapping, and degradation of active Si, which is consistent with its rapid capacity loss and low coulombic efficiency.

Overall, the d*Q*/d*V* analysis reinforces the earlier observations: BH-derived hard carbon acts as an effective host for Si, stabilizing the redox behaviour while suppressing interfacial degradation. Among all hybrids, the BH35–Si35 anode demonstrates the most stable reaction profile, reflecting its optimal balance between active material content and structural buffering capacity.

**Fig. 5:**
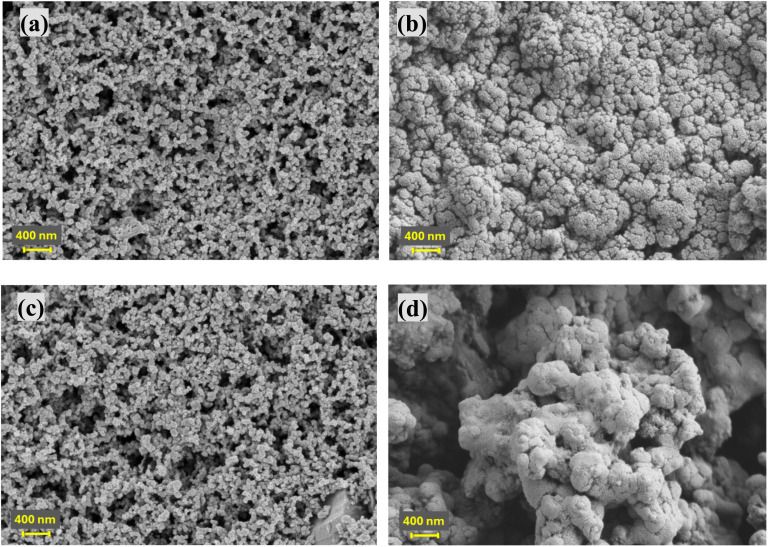
SEM images of the BH20–Si50 anode (a) before cycling and (b) after 100 cycles and Gr20–Si50 anode (c) before cycling and (d) after 100 cycles, all cycled at C/5.

To further investigate the structural and morphological stability of Si-containing anodes during cycling, SEM analysis was performed comparing the BH20–Si50 and Gr20–Si50 anodes before cycling and after 100 cycles (Fig. 5). Prior to cycling, both anodes display relatively homogeneous particle distributions with distinct morphologies. The BH20–Si50 anode exhibits a porous, irregular structure characteristic of biomass-derived carbon, while Gr20–Si50 presents a denser microstructure dominated by plate-like graphite flakes.

After 100 cycles, the BH20–Si50 anode retains notable structural integrity, retaining its porous architecture with only minor particle fragmentation and surface roughening. This observation confirms the effectiveness of the BH matrix in buffering the significant volume changes caused by repeated Si lithiation/delithiation, resulting in stable long-term cycling performance. In contrast, the Gr20–Si50 anode shows severe structural degradation after cycling, with extensive particle cracking, pulverization, and loss of the original morphology. The substantial morphological deterioration directly correlates with its rapid capacity fading observed previously. These morphological trends align with the impedance response, where BH–Si hybrids exhibit restrained resistance growth relative to Gr–Si and Si-only electrodes, indicating improved interfacial stability during repeated Si alloying/dealloying.

**Fig. 6:**
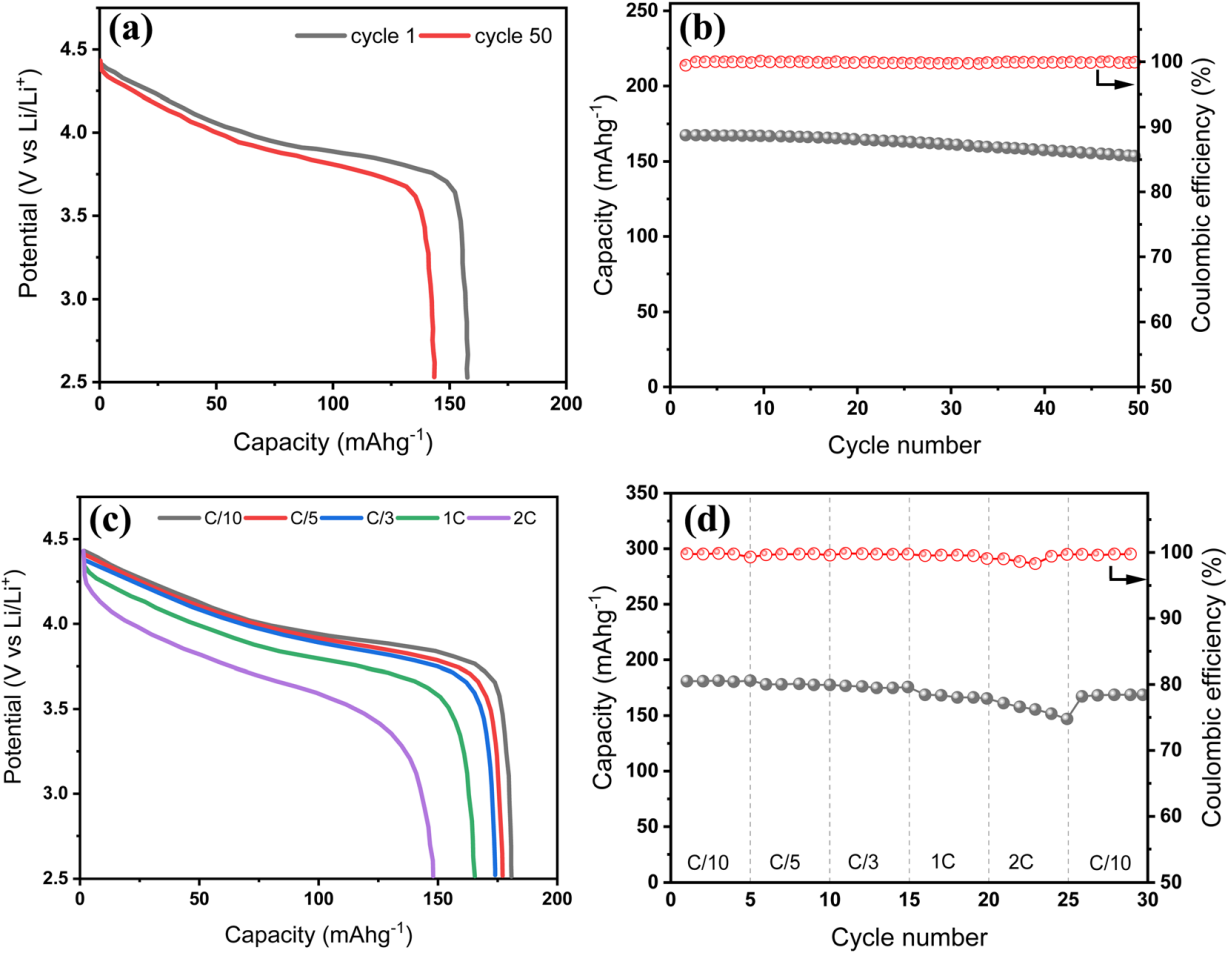
Electrochemical performance of NMC622 in half-cell configuration. (a) Voltage profiles at 1st and 50th cycles at C/5 current. (b) Cycling stability and coulombic efficiency at C/5 current. (c) Voltage profiles at various rates from C/10 to 2C. (d) Rate capability and capacity recovery.

To construct a full cell, we selected NMC622 as the cathode material due to its favourable balance of high capacity, structural stability, and cost-effectiveness. Its electrochemical performance was first evaluated in a half-cell configuration using Li metal as the counter/reference electrode, and the results are summarized in Fig. 6. As shown in Fig. 6a, the charge–discharge profiles at the 1st and 50th cycles display the characteristic sloping behaviour of layered oxide cathodes, with a well-defined voltage plateau centred at around 3.8 V *vs.* Li^+^/Li, consistent with reversible Li^+^ intercalation/deintercalation. The anode demonstrates robust cycling stability, with a capacity retention of 92% after 50 cycles at C/5 (Fig. 6b) and retains a high coulombic efficiency approaching 100% throughout cycling.

The rate capability results (Fig. 6c and d) further confirm the reliable kinetics of the NMC622 cathode. The voltage profiles remain smooth and consistent across increasing current rates, and the electrode retains 82% of its initial capacity when the rate is increased from C/10 to 2C. Importantly, full capacity recovery is observed when the current is returned to C/10, confirming good structural reversibility. These results validate NMC622 as a stable and high-performing cathode and support its integration into full-cell systems. Its reliable rate response and cycling retention make it an appropriate cathode for pairing with high-capacity anodes, such as the BH20–Si50 composite, in practical full-cell configurations.

**Fig. 7:**
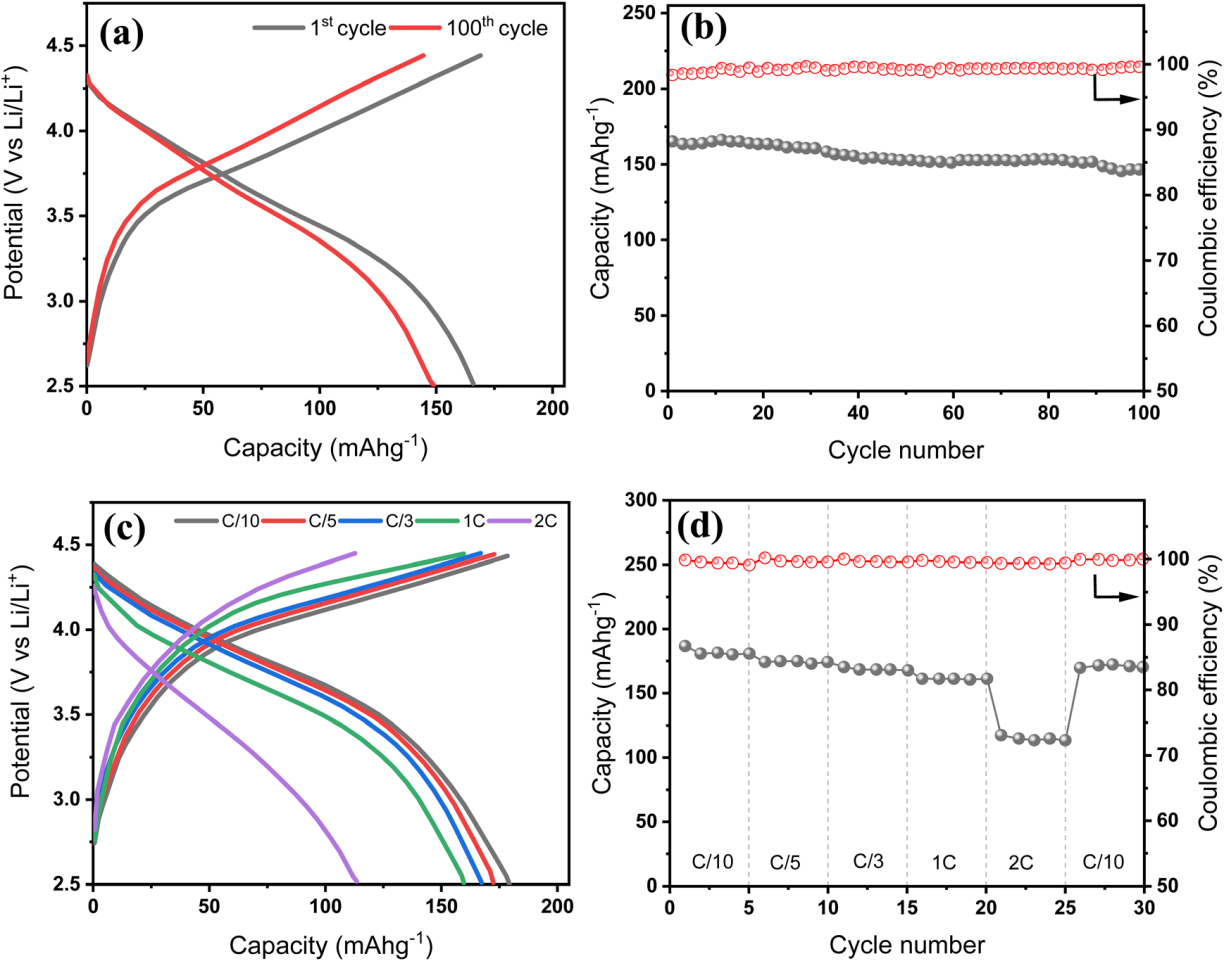
Electrochemical performance of the BH20–Si50//NMC622 full cell. (a) Charge–discharge voltage profiles at the 1st and 100th cycles, (b) cycling stability and coulombic efficiency over 100 cycles at C/5, (c) voltage profiles at varying current densities from C/10 to 2C, and (d) rate capability and capacity recovery.

To assess the practical applicability of the BH20–Si50 composite anode, full coin cells were assembled using NMC622 as the cathode and evaluated under various testing conditions. As shown in Fig. 7a, the voltage profiles at the 1st and 100th cycles exhibit typical sloping behaviour, with a discharge plateau near 3.6 V and a gradual increase in polarization over extended cycling—indicative of mild resistance growth and interfacial evolution. The cycling performance of the full cell is presented in Fig. 7b. At a current rate of C/5, the cell delivers an initial discharge capacity of 165 mAh g^−1^ (based on the cathode mass), with a high ICE of 98.3%. After 100 cycles, the discharge capacity remains at 147 mAh g^−1^, corresponding to a capacity retention of 89%, while the coulombic efficiency improves to 99.5%, reflecting stable Li utilization and effective SEI passivation across the cell. Fig. 7c and d show the voltage profiles and rate capability of the full cell at various current rates from C/10 to 2C. The cell retains ∼160 mAh g^−1^ up to 1C, corresponding to ∼90% rate capability, and recovers well when returned to C/10, indicating good structural reversibility and kinetic accessibility. At 2C, however, the capacity drops to 113 mAh g^−1^, reflecting a rate capability of 63%, likely due to increased kinetic limitations and interfacial polarization on both electrodes.

Based on the total active material and electrolyte mass, the estimated energy density of the full cell is calculated to be 385, 373, 361, 344, and 245 Wh kg^−1^ at C/10, C/5, C/3, 1C, and 2C, respectively. This performance enhancement over conventional graphite//NMC full cells can be attributed to the incorporation of Si into the biomass-derived carbon matrix, which provides mechanical buffering, enhances capacity, and effectively mitigates particle disintegration during repeated cycling. These results confirm the electrochemical compatibility of the BH20–Si50 anode with high-voltage cathodes and demonstrate its feasibility for practical Li-ion full-cell applications with high energy density and reliable cycling performance.

The electrode loading was selected as a consistent baseline that enables meaningful comparison between BH–Si hybrids, graphite-based composites, and Si-only electrodes under identical electrode-processing conditions. From a practical perspective, higher active material fractions are desirable to maximise energy density; however, increasing the active fraction typically reduces binder and conductive additive content, which can accelerate contact loss and exacerbate impedance growth in alloying-type anodes. Consequently, optimisation of the active material fraction and electrode engineering (binder system, porosity, calendering and areal loading) remains an important future step for translating BH–Si hybrids towards industrially relevant electrode designs.

## 3. Conclusion

In summary, we demonstrated that barley husk-derived carbon (BH) is an effective host material for accommodating high silicon (Si) loadings in LIB anodes. BH–Si composite anodes showed significantly enhanced electrochemical performance compared to graphite–Si counterparts, attributed to the structural and morphological stability provided by the BH matrix. Among various compositions tested, the BH20–Si50 composite exhibited remarkable cycling stability, retaining approximately 65.8% of its initial capacity after 500 cycles, alongside superior rate performance and improved structural integrity, as confirmed by post-cycling SEM analyses. A full cell pairing a BH20–Si50 anode with an NMC622 cathode achieved stable cycling with 89% capacity retention after 100 cycles and an impressive energy density of up to 385 Wh kg^−1^. These results highlight the potential of biomass-derived carbon matrices, particularly BH, as sustainable and structurally robust materials for high-performance, Si-based anodes in next-generation LIBs.

## 4. Experimental section

### 4.1. Material preparation

Barley husks were obtained from a local brewery near Norwich, United Kingdom. The raw barley husks were first ground into fine fragments and thoroughly washed with deionized (DI) water to remove surface impurities. The cleaned biomass was dried at 60 °C overnight in a conventional oven. For carbonization, 35 g of the dried barley husks was transferred to ceramic crucibles and subjected to pyrolysis in a tube furnace (Carbolite TZF 12/65/550) under a continuous nitrogen flow. The temperature was raised to 1150 °C at a rate of 5 °C min^−1^ and held for 2 hours. After natural cooling to ambient temperature, the carbonized product, containing both carbon and silica phases, was collected as a black powder (BHs-SiO_2_/C).

To improve homogeneity and enhance the electrochemical interface, the BHs-SiO_2_/C powder underwent mechanical ball milling using 1/2-inch stainless steel balls in a high-energy mixer mill (Spex 8000D) at 1400 rpm for 20 minutes. The milled powder was then immersed in 1 M hydrochloric acid (Merck) for 8 hours at room temperature to remove residual inorganic impurities. Following acid treatment, the mixture was vacuum filtered and washed repeatedly with DI water until neutral pH was achieved. The purified material was dried at 50 °C overnight, yielding the final barley husk-based active powder (denoted as BH).

To fabricate the silicon-enhanced anode materials, commercially available synthetic silicon powder (Siligrain e-Si 410 from Elkem, 99.8%, approximately 400 nm) was mechanically blended with the BH powder in three different mass ratios: 20 : 50, 35 : 35, and 50 : 20 (BH : Si). The resulting composites were denoted as BH20–Si50, BH35–Si35, and BH50–Si20, respectively. The mixtures were further homogenized by additional ball milling under the same conditions described above to ensure uniform dispersion of Si within the BH matrix.

For comparative analysis, additional anode compositions were prepared: a silicon-only anode (Si) containing 70 wt% synthetic Si; a binary mixture of graphite and Si (Gr20–Si50) with a 20 : 50 weight ratio; and two control anodes based solely on either barley husks (BH) or commercial graphite (Gr, Acros Organics, battery grade). The labels used for each anode configuration are summarized in Table 1 and will be used consistently throughout this manuscript for clarity and brevity.

**Table 1:** Composition and labels of prepared anode materials

Anode	Anode composition	Description
Gr	70% graphite + 20% C65 + 10% PAA	Conventional graphite control
BH	70% BH + 20% C65 + 10% PAA	Barley husk-based control
BH50–Si20	50% BH + 20% Si + 20% C65 + 10% PAA	BH-dominant hybrid
BH35–Si35	35% BH + 35% Si + 20% C65 + 10% PAA	Balanced BH and Si hybrid
BH20–Si50	20% BH + 50% Si + 20% C65 + 10% PAA	Si-rich hybrid
Gr20–Si50	20% graphite + 50% Si + 20% C65 + 10% PAA	Graphite-supported Si composite
Si	70% Si + 20% C65 + 10% PAA	Pure silicon anode

### 4.2. Characterization and measurements

Comprehensive structural and compositional characterization was carried out on the primary active materials—BH, Si, and Gr—to evaluate their morphological, crystallographic, and surface chemical properties prior to electrode fabrication. The morphology and particle distribution of the powders were examined *via* scanning electron microscopy (SEM, Zeiss Gemini 300 FE), while elemental mapping using energy-dispersive X-ray spectroscopy (EDX, Oxford Instruments Ultim Max 170) provided spatial distribution of carbon, silicon, and oxygen. X-ray diffraction (XRD, Rigaku Smartlab SE) with Cu Kα radiation (*λ* = 1.540 Å, 40 kV, 50 mA) was employed over a 2*θ* range of 10–80° to investigate crystallinity and phase composition.

Each electrode slurry was prepared by mixing the active material (70 wt%), carbon black C65 (20 wt%) as the conductive additive, and polyacrylic acid (10 wt%) as the binder, dispersed in DI water to form a uniform paste. The slurry was then cast onto copper foil substrates using a doctor blade with a wet film thickness of 200 µm, followed by vacuum drying at 80 °C for 12 hours. Dried electrodes were punched into 14 mm diameter disks (geometric area 1.54 cm^2^) with an areal mass loading of 1.04 mg cm^−2^ for cell assembly and electrochemical testing. All electrochemical evaluations were conducted using 2016-type coin cells assembled in an argon-filled glovebox (MBRAUN UNIlab Plus ECO) with both oxygen and moisture levels maintained below 0.5 ppm. Lithium metal chips were employed as the counter electrode, while a microporous polypropylene membrane (Celgard 2325) served as the separator. The electrolyte was purchased from Shenzhen Laborxing Technology Ltd and consisted of 1.2 M LiPF_6_ in a mixed solvent of ethylene carbonate (EC) and ethyl methyl carbonate (EMC) in a 1 : 3 volumetric ratio, supplemented with 15 wt% fluoroethylene carbonate (FEC) and 3 wt% vinylene carbonate (VC) as SEI-forming additives.

The assembled half-cells were allowed to rest for 24 hours at room temperature before testing. Galvanostatic charge–discharge measurements were performed using a BioLogic BCS-850 and LAND CT3001A battery tester within a voltage range of 0.01–3.0 V (*vs.* Li^+^/Li). The current density for 1C was defined based on the capacity of the active components: 1800 mAh g^−1^ for Si and 380 mAh g^−1^ for BH. The specific 1C current value for each sample was calculated according to the mass ratio of Si and BH in the electrode formulation. Note that C-rates were defined for each anode formulation using the reference capacities listed in Fig. S1; therefore, the corresponding applied current density (mA g^−1^) differs between electrodes. Cyclic voltammetry (CV) was carried out at a scan rate of 0.2 mV s^−1^ using a CHI 660E electrochemical workstation, and electrochemical impedance spectroscopy (EIS) was conducted over a frequency range of 100 kHz to 0.01 Hz with a 5 mV perturbation amplitude.

In addition to half-cell studies, full cells were assembled to further assess the practical applicability of the most promising anode compositions. Nickel manganese cobalt oxide (NMC622) was selected as the cathode. The full cells were balanced using a target N/P ratio of 1.05 (negative-to-positive capacity ratio). The full cells were configured in a cathode-limited manner, such that the negative electrode capacity slightly exceeded that of the cathode to mitigate lithium inventory loss associated with interphase formation and to reduce the risk of lithium plating during cycling. Full-cell assembly followed similar procedures under inert conditions, with the Si-rich hybrid anode paired against the NMC622 cathode using the same electrolyte and separator system.

Following electrochemical cycling, selected coin cells were disassembled inside an argon-filled glovebox. The anodes were carefully extracted, and any remaining electrolyte was gently rinsed off using anhydrous dimethyl carbonate (DMC, Sigma-Aldrich). The electrodes were then dried under inert conditions within the glovebox for 12–24 hours. No thermal treatment was conducted to retain the surface morphology for postmortem SEM analysis.

## Conflicts of interest

There are no conflicts to declare.

## Supplementary Material

NA-OLF-D5NA01100K-s001

## Data Availability

The data that support the findings of this study are available from the corresponding author upon reasonable request. Supplementary information (SI) is available. See DOI: https://doi.org/10.1039/d5na01100k.
