# Supplementary material for: Biomass-derived hard carbon host with added commercial silicon for high-capacity lithium-ion battery anodes
Source: Nanoscale Adv. 2026 Feb 23;8(7):2398–410. doi: 10.1039/d5na01100k (PMC12969071; doi:10.1039/d5na01100k)
Supplement: NA-008-D5NA01100K-s001 [file NA-008-D5NA01100K-s001.pdf]

Supplementary Information (SI) for Nanoscale Advances.
This journal is © The Royal Society of Chemistry 2026

Supplementary Information for

Biomass-Derived Hard Carbon Host with Added Commercial Silicon for High-Capacity Lithium-ion Battery Anodes

Alireza Fereydooni [a,b,c†], *Chenghao Yue* [a,b†], *Puritut Nakhanivej* [d], *Maria Balart Murria* [d], *Mingrui Liu* [a], *Yuexi Zeng* [a], *Zhijie Wei* [a], *Qiuju Fu* [e], *Xuebo Zhao* [e], *Melanie Loveridge* [d], *and Yimin Chao* [a,b]*

[a.] *National Energy Key Laboratory for New Hydrogen-Ammonia Energy Technologies, Foshan Xianhu Laboratory, Foshan 528200, P. R. China.*
[b.] *School of Chemistry, University of East Anglia, Norwich NR4 7TJ, UK.*
[c.] *Tyndall Center for Climate Change Research, University of East Anglia, Norwich NR4 7TJ, UK.*
[d.] *Warwick Manufacturing Group (WMG), University of Warwick, Coventry CV4 7AL, UK.*
[e.] *Shandong Provincial Key Laboratory of Chemistry Energy Storage and Novel Cell Technology, School of Materials Science and Engineering, Qilu University of Technology (Shandong Academy of Science), Jinan 250353, China.*

* Corresponding author's E-mail address: Y.Chao@uea.ac.uk

† These authors contributed equally to this work.

Keywords: anode materials, lithium-ion batteries, silicon, barley husks, sustainability

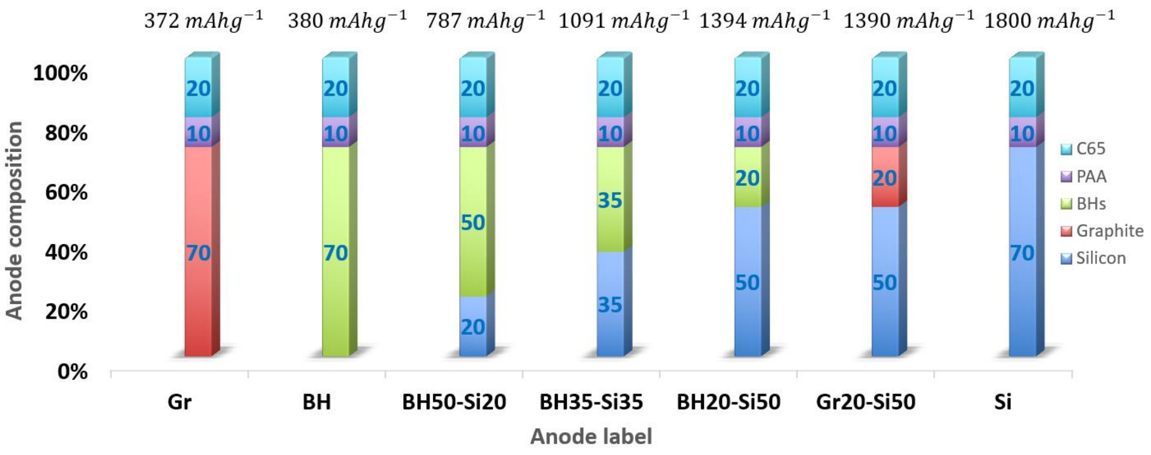

Figure S1. Composition of the investigated anodes (wt%) and the corresponding reference specific capacities used to define the C-rate for each formulation. All current densities reported in the manuscript were calculated from these 1C values.

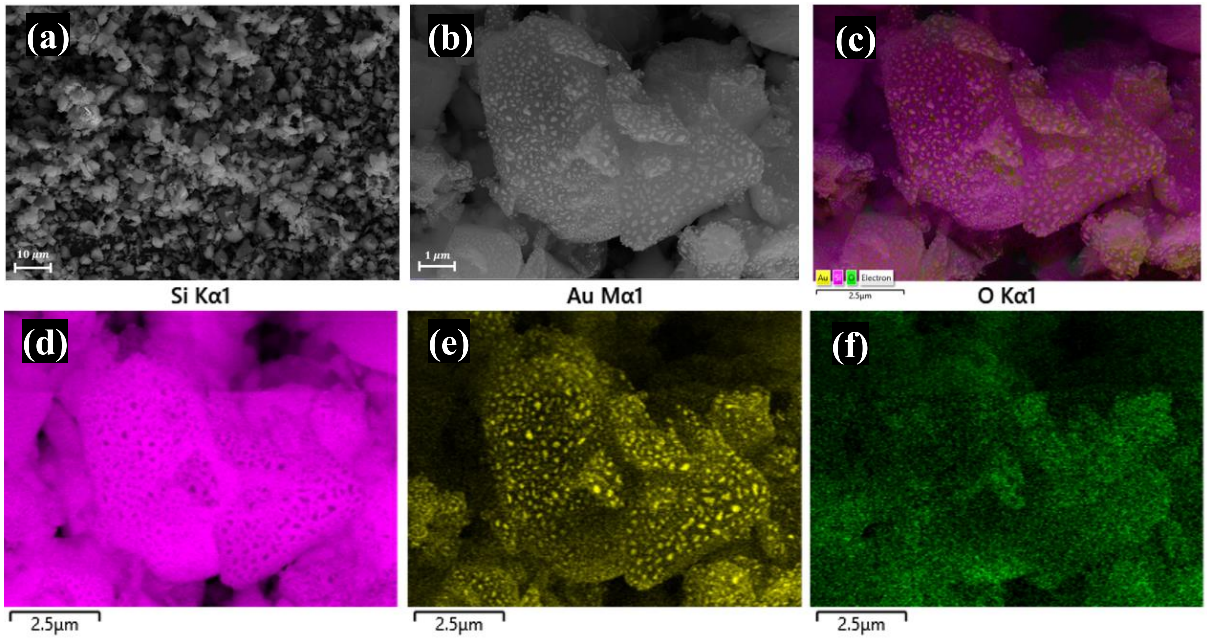

Figure S2. SEM and EDS characterisation of the commercial Si powder used as additive in the anodes. a) Low-magnification SEM image of Si powder, and b) to f) high-magnification SEM image and corresponding elemental maps for Si $K\alpha_1$, Au $M\alpha_1$ (sputter coating) and O $K\alpha_1$.

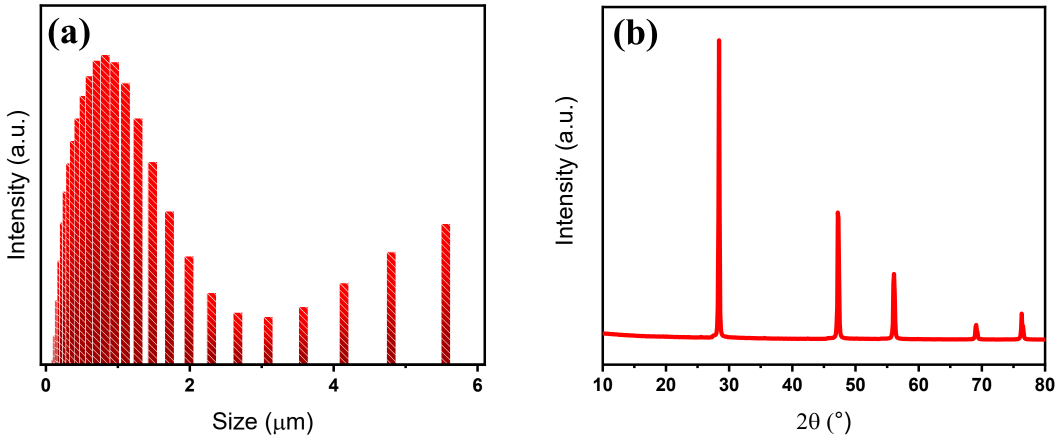

Figure S3. Structural and size characterisation of the commercial Si powder: a) particle-size distribution obtained by DLS, indicating a dominant population of sub-micrometre to few-micrometre Si agglomerates; b) XRD pattern indexed to crystalline Si, showing sharp reflections characteristic of diamond-cubic Si with no additional crystalline oxide phases.

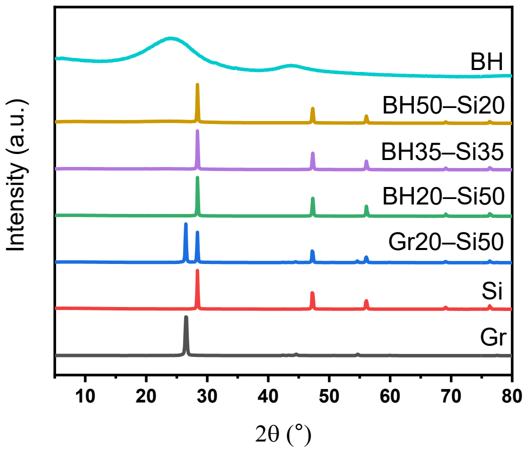

Figure S4. X-ray diffraction patterns of graphite (Gr), commercial silicon (Si), Gr20–Si50, BH-derived carbon (BH), and BH–Si hybrid anodes (BH50–Si20, BH35–Si35, BH20–Si50), showing crystalline Si reflections superimposed on the disordered carbon background.
